# Not Just a Headache: Qualitative Study About Web-Based Self-Presentation and Social Media Use by People With Migraine

**DOI:** 10.2196/10479

**Published:** 2019-06-19

**Authors:** Carly Pearson, Rosanna Swindale, Peter Keighley, Alison Ruth McKinlay, Leone Ridsdale

**Affiliations:** 1 King's College London Institute of Psychiatry, Psychology and Neuroscience London United Kingdom

**Keywords:** migraine, internet, social media, eHealth, social support, self-management, qualitative research

## Abstract

**Background:**

To help with a long-term but invisible medical condition such as migraine, many people seek information and support on social media. The effect of using social media for people with migraine is not fully understood and remains to be investigated.

**Objective:**

The aim of this study was to describe how people with migraine use social media and how social media use affects their identity and sense of self.

**Methods:**

A total of 20 participants who experienced migraine were recruited via migraine-specific charities. Semistructured interviews were conducted with questions based on a topic guide. Interviews were transcribed verbatim, and transcripts were analyzed using thematic analysis.

**Results:**

People with migraine are using social media to obtain information to better understand their condition and treatment options. Social media offers instant access to continuous information and social support. This exchange of social support and information was viewed as mutually beneficial. Participants viewed social media as an outlet to vent frustrations and validate the migraine experience. Several participants pointed out that the invisible and episodic nature of migraine can lead to societal misunderstanding of the impact and or severity of their condition. Some participants masked their online migraine-related behavior using different sites or closed online groups to control who saw their migraine-related content. Participating in closed social media groups sometimes changed Web-based behavior in other areas of the platform. This illustrates the complex relationship between migraine, social media, and identity.

**Conclusions:**

How migraine is part of an individual’s identity and how this is represented online can vary. Social media can provide people who experience migraine with instant and continuous access to support and information, from a group of empathic others with similar lived experiences. Social media is used to validate the illness experience, as well as provide reassurance and help reduce feelings of isolation.

## Introduction

Migraines are severe headaches that affect at least 5% of men and 15% of women [1]. Symptoms can include heightened sensitivity to external stimuli, nausea, vomiting, and sensory disturbances (auras) [1,2]. Between attacks, people with migraine display no physical symptoms and might choose to conceal their condition, known as *passing* [3-5]. Successful passing allows those with invisible illnesses to present themselves in ways that are not defined by their impairment [3]. Despite this, migraine is a condition that can affect identity and self-concept. Episodes can place burden directly on the individual but also have indirect effects on wider society. It is now the commonest cause of disability in people of working age [6].

Migraine symptoms are typically episodic and unpredictable, making it a difficult condition to manage in daily life. It is distinctive from other chronic conditions as it lacks a visible illness marker [7]. Kleinman proposed that such problems can cause frustration from not being believed or understood and result in altered self-concept or self-esteem [8]. People with migraine may not receive adequate support owing to the experience of illness and pain being subjective [7]. Others might not believe or understand the impact of migraine symptoms [9]. This group may therefore feel dissatisfied with their support and medical treatment [10-12], which can make the experience of receiving validation and acknowledgement from others especially valuable.

Living with a long-term condition requires self-management, where individuals take control of their health care through targeted symptom management and lifestyle changes [12]. Internet-based technologies offer a way to self-manage, and more recently, social media has enabled the rapid exchange of information [13] for this purpose. Social media includes internet-based websites and mobile phone apps that enable users to generate content and interact with others [14,15]. The growth of social media has meant that people with health conditions can rapidly acquire and share health-related information [16]. Researchers have called for further study into the precursors of lasting health-related change through the use of social media [17].

Social identity theory could explain some behavior change following Web-based interactions. If behaviors are linked to the role a person occupies within a social group (ie, son, parent, and teacher) [18], and social norms tied to group membership [19], this may apply to online group interactions as well. Under social identity theory [20], once an individual identifies with a social group, they may strive for a self-concept [18,21] associated with their group membership. In this study, we elected to focus on identity and presentation of *the self* among people with migraine in Web-based spaces. For the purposes of this discussion, the self is conceptualized as an accumulation of the personas that are represented in a broad set of social interactions [22].

Social media use has been reported to increase self-management in other long-term conditions such as diabetes [23-26]. However, there are likely to be differences in the perceived affordances of social media among medical conditions, and online self-presentation in migraine has yet to be investigated. Interest in the use of social media in migraine and how individuals perceive it is growing [13,27]. The principal investigator in this study is a neurologist who runs a headache clinic and evaluates health interventions designed to improve self-management using qualitative methods [28,29]. Funding from the European Research Council allowed the group to extend their research, aiming to explore how people with migraine independently use social media in the context of their lives and how this use impacts them.

Further exploration of behavior change following social media use in people with chronic long-term conditions is warranted [17]. One possible mechanism is that users must curate their identity and self-presentation using social media platforms. Therefore, we asked the following: What are the uses of social media among people with migraines? How does use of social media affect an individual’s sense of self and online identity?

## Methods

### Study Design

We used qualitative methodology aiming to gather in-depth views and experiences of using social media in the context of migraines. This approach permits open exploration of participants’ views, and the interviewer can adapt questioning based on each participant’s response. Ethical approval was given by the Psychiatry, Nursing and Midwifery Research Ethics Subcommittees, King’s College London (reference: PNM/13/14-18).

### Participants and Recruitment

Participants expressed interest in response to advertisements placed on social media sites and newsletters of the charities: Migraine Trust and Migraine Action. Inclusion criteria were adults aged ≥18 years (no upper age limit), self-report experience of migraines, use social media in the context of migraine, fluent in English, and live within a catchment area of London and Birmingham in the United Kingdom.

A total of 35 eligible participants responded from all over the United Kingdom. Participants were selected depending on their proximity to the place of work of the interviewers, which was London and Birmingham. They were then invited for an interview in their home or a public space near their homes. In total, 2 participants selected for the interview did not participate, one owing to work commitments and another was uncontactable. All participants received an information sheet explaining the aims and methods of the study and gave written informed consent to participate. Recruitment ended at 20 participants when data saturation was reached.

### Data Collection

One-to-one semistructured interviews were conducted by the researchers who were trained in qualitative methods with the experience of working with patients and research participants. The interviews lasted approximately 30 to 45 min. Interviews took place between November 2016 and March 2017. An interview schedule was devised with questions developed from the existing literature to explore the study aims (Table 1). Pilot interviews (P5, P10, and P19) were conducted initially to assess the viability of the interview schedule, and minor iterations were implemented to ensure clarity of the questions. This involved clarifying language in the question around identity and subsequent probes.

**Table 1:** Interview topics and probes.

Questions	Probes
When did you first start to experience migraine?	How did you feel when you first started to experience migraine?
Could you tell me what personal research have you done around migraine? (eg, family/friends, self-help books)	Do you know anyone else in real life who experiences migraine?
Could you talk about any social media platforms you have used specifically relating to migraine? (eg, Twitter, Facebook, blogging)	What were your motivations for you to visit this website or use this app?
Have you ever found information or support for your migraines on social media that you didn’t find elsewhere?	Has being part of the online migraine community impacted on your migraine management?
Could you describe how you use social media in the context of your migraine to find out new information and advice?	Do you use the official NHS^a^ choices website to gather information about migraine?
Could you describe how you use social media in the context of your migraine to share information and advice?	Would you say you primarily seek to find out information or to share it?
Have you found communicating online with others who have migraine a supportive experience?	Is the social support online different to offline?
Could you describe any benefits of having an online presence?	What can social media offer that other sources of information cannot?
Could you describe any drawbacks of having an online presence?	Do you find the information to be accurate and reliable?
Do you see social media platforms as a way of gaining expertise in migraine knowledge?	How does this compare to traditional methods of speaking one-to-one in person with a healthcare professional?
What kind of impact has using social media had on your identity?	How do your migraines fit into your online identity?
Have you ever had a bad reaction from other people in relation to experiencing migraines?	Has social media helped you to deal with this reaction?
Is there any way you would like to see social media platforms for migraine improved?	Potential areas: Convenience, quality, comprehensiveness, immediacy of information, user demographics, user uptake, privacy.
Would you recommend that other people who experience migraine use social media?	Could you explain why you would or wouldn’t recommend it?

^a^NHS: National Health Service.

### Data Analysis

Interviews were audio-recorded and transferred to a password protected computer. To preserve participant anonymity, identifying data were not transcribed. Audio and transcript files were identifiable only by an arbitrary participant number. Interviews were transcribed verbatim by a third-party transcription service and checked by interviewers. The transcripts were analyzed using thematic analysis which involves inductive interpretation [30,31]. No external sources other than participant transcripts were used during the analysis process. The transcripts were coded line-by-line using NVivo 11 software for qualitative data. Similar codes were grouped together, and an iterative approach was used to create subthemes. These were further grouped together into overarching themes. A discussion of the themes and interpretations was conducted by all authors. The final interpretation of codes and themes was completed by the senior researchers (CP and LR).

## Results

### Participant Characteristics

Of the 20 participants interviewed, 17 were women and all were white (Table 2). The age range was 24 to 59 years, mean 39 years. Almost three-quarters (70%) had graduate or postgraduate level education. A total of 11 participants were in full-time employment and 6 were unemployed. All reported a diagnosis of migraine, with the frequency ranging from 1 day per month to daily (mean 12 days per month).

### Social Media Use

Facebook was the most commonly used social media platform (19 participants). Many sought information and social support from closed migraine-specific Facebook groups. Blogs were used by 2 participants. A total of 3 participants used YouTube to observe migraine symptoms and learn about migraine causes or pain reduction. One participant created YouTube videos to educate others about migraine. Twitter was often viewed as a more professional domain rather than personal. Participants’ use of social media tended to fit on a spectrum from actively engaging with social media to more observational social media users, and some people in between.

**Table 2:** Participant demographics.

Participant	Sex	Age (years)	Highest qualification	Employment	Living situation	Migraine days per month (n)	Social media sites used in context of migraines
P1	Female	31	Postsecondary	Unemployed	With others	3	F^a^, T^b^, I^c^
P2	Female	52	Postsecondary	Full-time	Alone	12	F
P3	Female	28	Postgraduate	Student	With others	2	F, T, Y^d^
P4	Female	35	Postgraduate	Part-time	With others	9	F, T, Y, B^e^
P5	Female	31	Unknown	Employed	With others	8	F
P6	Male	43	Postsecondary	Full-time	With others	4	F, T
P7	Female	23	Postgraduate	Full-time	With others	16	F, I
P8	Male	24	Postgraduate	Student	With others	1-2	F, T
P9	Male	25	Postsecondary	Unemployed	With others	1-2	F, Y
P10	Female	47	Graduate	Unemployed	With others	29	F, M^f^, V^g^
P11	Female	29	Postgraduate	Full-time	With others	1	F, I
P12	Female	45	Postgraduate	Full-time	With others	12	F, P^h^
P13	Female	33	Graduate	Full- time	With others	10-15	Y, I
P14	Female	32	Postgraduate	Part-time	With others	Daily	F
P15	Female	58	Postgraduate	Part-time	With others	5	F, H^i^, B, MT^j^, Migraine Buddy app
P16	Female	31	Graduate	Unemployed	With others	5	F
P17	Female	59	Graduate	Retired	With others	15	F, I
P18	Female	54	Postsecondary	Unemployed	With others	3-5	F
P19	Female	47	Postsecondary	Part-time	With others	15	F
P20	Female	55	Postgraduate	Unemployed	With others	Daily	F

^a^F: Facebook.

^b^T: Twitter.

^c^I: Instagram.

^d^Y: YouTube.

^e^B: blogs.

^f^M: Migraine.com (information website with discussion facilities).

^g^V: Vestibular migraine website with online community.

^h^P: Pinterest.

^i^H: Health Unlocked (online social health network).

^j^MT: My Migraine Team (a social network for people with migraine).

### Theme 1: Information Exchange

#### Seeking Information

All participants used social media to obtain information about migraine. In total, 11 said they gained expertise in migraine knowledge and 13 gained awareness of new treatments. For some, interacting with others in Facebook groups resulted in a change of self-management for migraines. For example:

I follow some online Facebook groups or pages on politics and health policies. And that’s really helpful...in fact it was through that, that I pursued TMS [Transcranial Magnetic Stimulation].

P10

...you can go back to your doctor, or your specialist or whatever, and say, “I haven’t tried this one,” or, “People have said that this works in conjunction with that,” or whatever, “What do you think?”

P18

#### Sharing Information

In total, 14 participants said they shared information with other users on social media, mostly on Facebook. Some shared information with others who experience migraines in migraine-specific groups:

If I see an article that I think would be useful or a diagram or something which I think would be useful to other groups I would usually share it.

P20

Others used social media to share information with people who do not experience migraine by posting on their personal Facebook *walls*, seeking to help others to understand their condition:

I just bombarded all my family and friends with every article I could find...this is what’s happening to me.

P16

#### Pooling and Exchanging Information and Experiences

In total, 16 participants spoke about the benefit of pooling knowledge on social media. The dialogue and collaboration with other users added another level of benefit to information seeking and sharing:

...what used to happen before social media, you would research it by just Googling it and then wading your way through different articles or going to different doctors. Whereas now you can be part of a group and get first-hand experience and knowledge.

P18

A total of 12 participants said information gathered on social media had increased their confidence, knowledge, and skills in managing their own health care:

It’s given me information, like, because I might mention a side effect for a drug, and the doctor might be like, “Oh, I’ve not heard anyone having that.” And I’ll be like, “Well, actually, I’ve spoken to various people on Twitter and they all have it,” so it gives me a bit more, sort of, clout with what I’m saying...Which otherwise...you can feel very, like the only one experiencing that.

P4

Others benefited from reading the online discussion and not taking part themselves, whereas 8 participants referred to the benefit of sharing advice:

...[sharing advice] gets away from the feeling that we’re just an emotional support group. No, we’re not, we can actually give people the information that they need to make their lives easier and that feels good.

P20

### Theme 2: Social Support

#### Reducing Isolation

A total of 10 participants described not feeling alone and that social media had helped them to feel less isolated:

It’s comforting to know that I’m not on my own.

P2

In total, 9 participants spoke of having to cancel plans owing to their migraines. For some, social media provided a source of support for an unpredictable and invisible illness:

...when you cancel on them [offline contacts] for the fifth time you can see after a while it starts grating and rubbing on them...Then when you’ve got like a group that you can go to...and say, “I’ve got a migraine coming on again,” or whatever, you don’t get, “Oh, you know, you let me down last week,”...You get sympathy and empathy and understanding and recommendations and help which is nice.

P18

#### Reassurance

For 19 participants, the process of being able to hear about others’ experiences and compare them with their own provided a sense of comfort:

It is always more reassuring when their story fits your story.

P9

In the cases where people were unsure of what they were experiencing, reading similar accounts from others provided validation and reminded them that they were not alone. After accessing content on social media, some participants benefited from reassurance regarding *unusual* symptoms:

...on YouTube...People have made videos of how their...aura then kind of grows and disappears, and I’ve sort of watched it...to get reassurance that this isn’t me, sort of, going a bit mad...other people experience similar symptoms.

P3

In this sense, the use of social media served to normalize what some felt might be abnormal. In total, 6 participants also described social media as a *lifeline*:

I don’t know how people survived beforehand actually, especially because it’s [migraine] invisible.

P20

#### Forming a Continuous Support Group

A total of 10 participants referred to social media being available all the time, providing a continual source of contact with other users. In total, 7 participants described how social media groups can act as a support group:

...you can do it anytime, it’s not like a traditional support group where you’d have to go, you’d have to wait a week or a month...

P14

A total of 9 participants said that offering social support can give a reciprocal benefit:

...it’s mutually supportive because you can help them which in turn makes you feel better...

P18

Social media was used to gain access to an empathetic audience that is not limited to geographic location:

...so I went on to Facebook and I put hemiplegic migraine and I found this amazing support group of people.

P16

### Theme 3: Validation of Migraine Experience

#### Understanding Migraine

A total of 14 participants referred to migraine being an invisible illness, with 10 participants saying they had been given patronizing or unhelpful advice offline by others who often saw migraine as *just a headache*. In total, 18 participants discussed how the use of social media can help validate the migraine experience and combat the lack of understanding about the unpredictable and invisible nature of migraine:

I think the worst thing for people is not getting support...I think social media can be a good way of calling that out when we see it and people going: “Yes, that happened to me. That’s not okay.” There’s quite a lot of validating involved.

P20

I think it’s [social media] made me feel a bit better myself, really in that...It [migraine] is a genuine thing. It’s not just something that you bring on yourself because you are a wimp...

P17

#### The Value of Lived Experiences of Others

In total, 16 participants discussed the dichotomy between subjective experience versus verified facts and medical knowledge. Although medical fact seeking was important, there was a desire to hear about the subjective experience of others:

[I look on social media]...60% for the objective stuff...and then 40% the experiences, because the thing with migraines is sometimes it is subjective...the fact that you hear it in, like, a story, as well, if it’s like a personal narrative, it’s a bit better than...a chart saying...a percentage point did this...

P8

A total of 10 participants spoke about the need to experience migraine to truly understand it. Being able to read the lived experiences of other people with migraine was beneficial in providing personally relevant information.

I mean, my partner’s very supportive. But again, there’s only so far he can empathize...when I am poorly, because he doesn’t know what it’s like, he hasn’t experienced one before.

P3

#### Catharsis

A total of 10 participants described how they used social media as an outlet for discussing frustrating migraine experiences. Social media was a resource for some participants to cope with the emotions that built up from their experiences:

I use it as a tool to cope as it’s quite useful for venting.

P10

In total, 8 participants described how venting to other people on social media can prevent over-burdening family and friends:

If you have a chronic condition...you don’t want to bore people absolutely rigid. So it’s really nice having that set of strangers who you can actually offload to away from your ordinary life...

P15

Another participant described sharing her migraine experience online as *therapeutic*, although 3 participants spoke about how using humor provided some light relief:

...one thing that it [Migraine Action Facebook page] had on there was some jokes about migraine...that was quite good, to actually share just silliness about it because sometimes you just have to laugh about it.

P17

### Theme 4: Presentation and Perception of the Self

#### Self-Awareness and Awareness of Others

A total of 5 participants described how their identity had evolved since beginning to use social media. This included changes in the way they presented themselves online, as well as changing views of social media uses:

[Migraines] weren’t part of my identity online because I didn’t want to admit that they were a thing...I didn’t want them to exist in my life, even though they did and they existed a lot. So that shift in mind-set...bringing them into the conversation...was quite important to me that I was just a bit more attentive on my own identity...

P13

In total, 8 participants described how they had different Web-based identities, and some had multiple profiles, often to avoid disclosure of their migraine experience in certain domains of their lives. For some, social media groups had influenced their Web-based behavior and they displayed a different *self* in a private or closed migraine forum, compared with on their personal Facebook timeline:

...I haven’t posted much at all on my own timeline, it’s more on the groups. So I have a different persona on the groups [closed migraine-specific Facebook groups]. “I’m in pain, please help. Having a rubbish day.” All that sort of stuff goes on that group, or the groups, and then I tend to put a braver face on to what’s going on in everyday life.

P18

A total of 5 participants were concerned about how others perceived them on social media, and for some, this affected how they presented themselves on Web-based media:

I will put things on there [the closed group] because I wouldn’t want my friends and my family to think, “Oh my God, she’s going on about migraine again.” So I like the fact that it’s not public to everybody else.

P15

#### Online and Offline Identity

Several participants alluded to the temporal nature of migraines and how the extremity of acute symptoms could greatly impact their sense of identity and self:

...when I’m like well...I’m just somebody who suffers with migraines, whereas when it’s extreme, I just feel like I am a migraine.

P16

There was also variation in how participants felt social media reflected their migraine experiences. One participant said using Twitter had helped them to continue their role as a knowledgeable scientist after having to leave their job owing to their migraines:

it’s helped me retain my identity, I guess, after I got sick.

P4

Another participant described how social media has helped her to normalize and accept her migraine experience:

...the tips I’ve got have helped me reduce my migraine, to hopefully shove migraine more out of my identity. But now, I don’t know whether it is almost desensitization because it comes up on my phone, on the newsfeed, at any time. So that makes it more part of normal life rather than being associated with an acute attack. Maybe in a way it helped it become less of my identity...

P15

## Discussion

### Principal Findings

Our findings suggest that people with migraine are using social media to obtain health-related information to better understand their condition and treatment options. Social media can offer instant access to continuous migraine-related information, as well as social support from empathic others. The opportunity to pool the subjective lived experience of migraines on social media was described as invaluable, and the exchange of support and information was viewed as mutually beneficial.

Some participants viewed social media as an outlet to vent frustrations and validate migraine experiences with other users. They spoke of the invisible and episodic nature of the condition that may contribute to societal misunderstanding about the impact and severity of migraine. Some participants masked their migraine-related behavior using different Web-based sites or closed social media groups to control who saw their migraine-related content. Utilizing social media in this way had enabled participants to retain desired aspects of their identity, depending on how the individual chose to engage with the platform.

### Limitations

This study was conducted with a user group of volunteers in 1 country. People who belong to user groups are likely to be more highly educated, just as our volunteers were highly educated people, and are not representative of the whole population. The study also relied on a self-report migraine diagnosis. Future research could sample a broader demographic and use a clinical migraine diagnosis.

### Comparison With Previous Work

Many participants said that the information they had accessed on social media enabled them to be more active in their own health care, altering their migraine management and increasing their confidence when interacting with health care professionals. For example, some participants requested treatments that they had learnt about on social media. Increased feelings of control and self-management following social media use have been observed elsewhere in other chronic conditions such as diabetes and hypertension [26,32].

Participants had varied affordances for social media sites. There was a continuum, with more engaged users creating and sharing content (eg, blogging and creating YouTube videos) and those who tended to observe rather than contribute themselves. There was also a group of mid-range users who engage with migraine-specific groups. We have previously observed similar Web-based affordances among people with epilepsy [33].

Benefits of social media use are mostly attributable to social support via Facebook and blogs [34]. Blogging was much less commonly observed in our sample, but Facebook was the most used site. The closed group function on Facebook seemed to create a setting by which people with migraine could overcome invalidation or misunderstanding by identifying with others in the group with whom they had shared experiences. Sharing ideas and information and accessing support within groups were the most common themes in our study, suggesting users benefited from the membership afforded particularly by private social media groups.

### Web-Based Migraine Information and Support Group

For some, use of social media for health care purposes may represent an unfulfilled need with their existing care and support networks [35]. The migraine experience is often misunderstood, which can cause isolation and loneliness. The temporality of the condition may restrict an individual’s ability to carry out daily activities and participate in their social life. Thus, social media groups are beneficial to those with unpredictable or episodic health conditions, as the support offered is not in a fixed temporal or spatial context. These are key benefits of Web-based support networks that are often restricted in *real life* support group settings [36-38].

An episodic and invisible condition can be difficult to understand by those who do not experience it [3]. Gaining support and information from those with a similar lived experience appears to be a typical use of social media. Overcoming a sense of being alone and sharing experiences have been described as a benefit of attending a self-management group course for people with epilepsy, another invisible, common episodic condition [39]. In a diabetes study about social support from computer-mediated environments, users were more able to engage with their peers and access individualized support tailored to their specific situation [36]. Our findings in migraine are consistent with this.

There is also scope for medical professionals to utilize social media as a health resource, for example, integrating patient data such as electronic diaries in routine practice [40]. Researchers suggest that platforms such as Twitter have the potential to be used for gathering service user feedback [41]. Both user groups and health care providers could learn from this feedback to improve the services and information they provide. Further exploration on the role of health care service providers’ use of social media is warranted [41].

### Validation of Migraine Experience

Participants spoke about how using social media helped acknowledge their experience of migraine. Migraine is often misclassified or compared with commonplace headache, making migraine seem even more invisible [7]. Other conditions such as chronic fatigue syndrome can also be incorrectly compared with the prevalent state of tiredness. Chronic fatigue patients and fatigued employees reported experiencing more negative interactions with co-workers and insufficient social support compared with people in cancer remission and healthy co-workers [42]. This is perhaps due to lay misconceptions about the condition, lack of clear external illness markers, and parallels drawn with other common conditions. These ideas of similarity challenge the individual with an invisible illness to explain their impairments to feel understood [3]. Thus, the experience of having migraine acknowledged and validated by other users on social media is particularly valuable.

The prevailing opinion that headache is normal and can be alleviated by a tablet may be a barrier to individuals with migraine obtaining adequate support and understanding [32]. Participants in our study often referred to their offline contacts giving them patronizing or unhelpful advice about treating migraines. The sudden onset of migraine along with unpredictable frequency and duration affects people’s social, domestic, and professional lives [9,10,43,44]. Some people report feeling that their partner [9] and those in their social network [10] do not believe the impact or severity of their headaches. Associating with a group of others online with similar experiences was described positively by participants in this study, resulting in empowerment and changes in self-management. Social media offered an easily accessible source of understanding from others with a similar lived experience, which offered the added benefit of not overburdening offline support networks.

### Identity and Self-Presentation in Migraine

Social context impacts the self that is presented at a family gathering, job interview, or romantic date [5], which is also true of Web-based self-presentation [45]. Goffman compared self-presentation to a stage performance, where the individual chooses what to present to the audience onstage and what to keep private backstage [5]. Self-presentation is likened to a personal exhibit, where individuals act as curators by choosing what to display to their audience [46]. Participants gave varied responses about how migraine fits with their sense of self and self-presentation. Several chose different forms of self-presentation depending on their role on social media, such as being an educator by sharing information with others.

Twitter users have cited their ideal audience on social media to be a mirror image of themselves [45]. Festinger suggested that individuals find their own opinions and personal beliefs more valid when they are shared to a sufficient degree of similarity with a group of others [47]. This desire for similarity may also explain the desire to connect with groups of other users with migraine symptoms. Such connections appeared to empower those who sought them. Our findings indicate that participants engaged with social media as a means of identifying with other users with migraine as a means of talking about issues that they may not have been able to discuss with people outside of these groups. This could be explained using the social identity theory [26], whereby membership to Web-based groups such as this are associated with positive feelings and behaviors [48] within these Web-based communities.

Through such groups, users can share aspects of themselves that they may not do so freely with others in their typical social circles. In part, stigma or a lack of understanding from others may further influence this user behavior in Web-based settings. Belonging to Web-based communities that offer membership based on shared characteristics may help users to form a coherent sense of self that is the same across online and offline encounters [49]. In this way, the access to Web-based groups with positive health-related characteristics may be especially useful to people with conditions requiring support and self-management.

### Conclusions

In this study, we broadly enquired how participants with migraine used social media platforms and how this group presented their identity online. Participants described cognitive and affective benefits owing to the use of social media, particularly, gathering and sharing information, as well as receiving ongoing support and validation. They described learning about new therapies and side effects that had helped them in consultations with doctors. Participants particularly valued the affordances provided by Facebook groups.

Similar to others with potentially stigmatizing conditions, people with migraine want empathy and understanding for their subjective, but real, disability [6,39]. Based on our findings, participants described protecting themselves, or this aspect of their identity, from people who might disparage or reject them because of their condition. The apparent secrecy offered by social media helped them come to terms and cope with their condition, while simultaneously and separately managing the rest of their lives.

Social media can help validate the experience of migraine and in turn help people who experience migraines to feel better understood and less alone. How migraine is part of a person’s identity and represented online varies. Further understanding about the needs of people with long-term chronic conditions may help in the development of future Web-based interventions to improve health and well-being. Activity that occurs in closed groups helps people to accept and manage their condition in a separate but synergistic way.

Official Institutions for further information on migraine are as follows: International Headache Society [50], European Headache Federation [51], American Migraine Foundation [52], American Pain Society [53], European Headache Alliance [54], National Headache Foundation [55], Migraine Trust [56], Migraine Action [57].

## Abbreviations

B: blogs
F: Facebook
H: Health Unlocked
I: Instagram
M: Migraine.com
MT: My Migraine Team
NHS: National Health Service
P: Pinterest
T: Twitter
V: Vestibular migraine website with online community
Y: YouTube

## References

[1] Weatherall MW (2015). The diagnosis and treatment of chronic migraine. Ther Adv Chronic Dis.

[2] Goadsby PJ, Sprenger T (2010). Current practice and future directions in the prevention and acute management of migraine. Lancet Neurol.

[3] Lingsom S (2008). Invisible impairments: dilemmas of concealment and disclosure. Scand J Disability Res.

[4] Goffman E (1990). Stigma: Notes On The Management Of Spoiled Identity.

[5] Goffman E (1990). The Presentation Of Self In Everyday Life.

[6] Steiner T, Stovner L, Vos T, Jensen R, Katsarava Z (2018). Migraine is first cause of disability in under 50s: will health politicians now take notice?. J Headache Pain.

[7] Lonardi C (2007). The passing dilemma in socially invisible diseases: narratives on chronic headache. Soc Sci Med.

[8] Kleinman A (1988). The Illness Narative: Suffering, Health and the Human Condition.

[9] Buse D, Scher A, Dodick DW, Reed ML, Fanning KM, Manack Adams A, Lipton RB (2016). Impact of migraine on the family: perspectives of people with migraine and their spouse/domestic partner in the CaMEO study. Mayo Clin Proc.

[10] Malone C, Bhowmick A, Wachholtz A (2015). Migraine: treatments, comorbidities, and quality of life, in the USA. J Pain Res.

[11] Bigal M, Serrano D, Reed M, Lipton R (2008). Chronic migraine in the population: burden, diagnosis, and satisfaction with treatment. Neurology.

[12] Peters M, Abu-Saad H, Vydelingum V, Dowson A, Murphy M (2004). Migraine and chronic daily headache management: a qualitative study of patients' perceptions. Scand J Caring Sci.

[13] Nascimento T, DosSantos M, Danciu T, DeBoer M, van Holsbeeck H, Lucas S, Aiello C, Khatib L, Bender MA, Zubieta JK, DaSilva AF, UMSoD (Under)Graduate Class of 2014 (2014). Real-time sharing and expression of migraine headache suffering on Twitter: a cross-sectional infodemiology study. J Med Internet Res.

[14] Kaplan A, Haenlein M (2010). Users of the world, unite! The challenges and opportunities of social media. Bus Horiz.

[15] Boyd D, Ellison N (2007). Social network sites: definition, history, and scholarship. J Comput-mediat Comm.

[16] Hawn C (2009). Take two aspirin and tweet me in the morning: how Twitter, Facebook, and other social media are reshaping health care. Health Aff (Millwood).

[17] Moorhead S, Hazlett D, Harrison L, Carroll J, Irwin A, Hoving C (2013). A new dimension of health care: systematic review of the uses, benefits, and limitations of social media for health communication. J Med Internet Res.

[18] Tajfel H, Turner J, Jost J, Sidanius J (2004). The social identity theory of intergroup behavior. Political Psychology: Key Readings.

[19] Hogg M, Reid S (2006). Social identity, self-categorization, and the communication of group norms. Commun Theor.

[20] Tafjel H, Turner TJ, Austin WG, Worchel S (1979). An integrative theory of intergroup conflict. The Social Psychology of Intergroup Relations.

[21] Tajfel H (1974). Social identity and intergroup behaviour. Soc Sci Inf.

[22] Brivio E, Ibarra FC (2009). Self presentation in blogs and social networks. Stud Health Technol Inform.

[23] Mosen D, Schmittdiel J, Hibbard J, Sobel D, Remmers C, Bellows J (2007). Is patient activation associated with outcomes of care for adults with chronic conditions?. J Ambul Care Manage.

[24] Lorig K, Holman H (2003). Self-management education: history, definition, outcomes, and mechanisms. Ann Behav Med.

[25] Merolli M, Gray K, Martin-Sanchez F (2013). Health outcomes and related effects of using social media in chronic disease management: a literature review and analysis of affordances. J Biomed Inform.

[26] Grosberg D, Grinvald H, Reuveni H, Magnezi R (2016). Frequent surfing on social health networks is associated with increased knowledge and patient health activation. J Med Internet Res.

[27] Linnman C, Maleki N, Becerra L, Borsook D (2013). Migraine tweets - what can online behavior tell us about disease?. Cephalalgia.

[28] Morgan M, Jenkins L, Ridsdale L (2007). Patient pressure for referral for headache: a qualitative study of GPs' referral behaviour. Br J Gen Pract.

[29] Morgan M, Cousins S, Middleton L, Warriner-Gallyer G, Ridsdale L (2015). Patients' experiences of a behavioural intervention for migraine headache: a qualitative study. J Headache Pain.

[30] Ritchie JS, Spencer L, O'Connor W, Ritchie JS, Lewis J (2003). Carrying out qualitative analysis. Qualitative Research Practice: A Guide For Social Science Students And Researchers.

[31] Braun V, Clarke V (2006). Using thematic analysis in psychology. Qual Res Psychol.

[32] Allen C, Vassilev I, Kennedy A, Rogers A (2016). Long-term condition self-management support in online communities: a meta-synthesis of qualitative papers. J Med Internet Res.

[33] McKinlay A, Ridsdale L (2018). Views of people with epilepsy about web-based self-presentation: a qualitative study. Interact J Med Res.

[34] Patel R, Chang T, Greysen S, Chopra V (2015). Social media use in chronic disease: a systematic review and novel taxonomy. Am J Med.

[35] Smailhodzic E, Hooijsma W, Boonstra A, Langley D (2016). Social media use in healthcare: a systematic review of effects on patients and on their relationship with healthcare professionals. BMC Health Serv Res.

[36] Lewinski A, Fisher E (2016). Social interaction in type 2 diabetes computer-mediated environments: how inherent features of the channels influence peer-to-peer interaction. Chronic Illn.

[37] Donath J (2007). Signals in Social Supernets. J Comput Mediat Comm.

[38] Linnman C, Maleki N, Becerra L, Borsook D (2013). Migraine tweets - what can online behavior tell us about disease?. Cephalalgia.

[39] Ridsdale L, Philpott S, Krooupa A, Morgan M (2017). People with epilepsy obtain added value from education in groups: results of a qualitative study. Eur J Neurol.

[40] Allena M, Cuzzoni M, Tassorelli C, Nappi G, Antonaci F (2012). An electronic diary on a palm device for headache monitoring: a preliminary experience. J Headache Pain.

[41] Shepherd A, Sanders C, Doyle M, Shaw J (2015). Using social media for support and feedback by mental health service users: thematic analysis of a twitter conversation. BMC Psychiatry.

[42] Prins J, Bos E, Huibers MJ, Servaes P, van der Werf SP, van der Meer JW, Bleijenberg G (2004). Social support and the persistence of complaints in chronic fatigue syndrome. Psychother Psychosom.

[43] Young W, Park J, Tian I, Kempner J (2013). The stigma of migraine. PLoS One.

[44] Cooke L, Becker W (2010). Migraine prevalence, treatment and impact: the Canadian women and migraine study. Can J Neurol Sci.

[45] Marwick A, boyd D (2010). I tweet honestly, I tweet passionately: Twitter users, context collapse, and the imagined audience. New Media Soc.

[46] Hogan B (2010). The presentation of self in the age of social media: distinguishing performances and exhibitions online. Bull Sci Technol Soc.

[47] Festinger L (1950). Informal social communication. Psychol Rev.

[48] Hogg M, Terry D, White K (1995). A tale of two theories: a critical comparison of identity theory with social identity theory. Soc Psychol Q.

[49] Manago A, Graham M, Greenfield P, Salimkhan G (2008). Self-presentation and gender on MySpace. J Appl Dev Psychol.

[50] International Headache Society.

[51] European Headache Federation.

[52] American Migraine Foundation.

[53] American Pain Society.

[54] European Headache Alliance.

[55] National Headache Foundation.

[56] Migraine Trust.

[57] Migraine Action.

